# Diffuse Large B-Cell Lymphoma Presenting With Isolated Splenomegaly in a 42-Year-Old Female: A Rare Incidence of Primary Splenic Involvement

**DOI:** 10.7759/cureus.101438

**Published:** 2026-01-13

**Authors:** Meena LN, Steve Thomas, Sri Gayathri Shanmugam

**Affiliations:** 1 General Medicine, Sri Ramachandra Institute of Higher Education and Research, Chennai, IND; 2 Haematology, Sri Ramachandra Institute of Higher Education and Research, Chennai, IND; 3 Pathology, Sri Ramachandra Institute of Higher Education and Research, Chennai, IND

**Keywords:** diffuse large b-cell lymphoma, isolated splenomegaly, primary splenic lymphoma, r-chop, spleen

## Abstract

Primary splenic involvement by diffuse large B-cell lymphoma (DLBCL) is uncommon and may present with nonspecific clinical features, making diagnosis challenging. We report the case of a 42-year-old female who presented with gradually increasing abdominal distension and weight loss over three months, without fever, night sweats, or lymphadenopathy. Examination revealed massive splenomegaly, while routine laboratory tests were unremarkable. Ultrasound and CT imaging demonstrated isolated splenomegaly, and PET-CT showed increased metabolic activity confined to the spleen. Bone marrow biopsy did not reveal any diagnostic abnormalities. The patient underwent diagnostic splenectomy, and histopathology confirmed DLBCL of the non-germinal center B-cell subtype. Immunohistochemistry was positive for CD45, CD20, BCL2, and MUM1, with a Ki-67 index of 60%. She received six cycles of R-CHOP (rituximab, cyclophosphamide, doxorubicin, vincristine, and prednisone) chemotherapy, which she tolerated well. Post-treatment PET-CT revealed a complete metabolic response, and subsequent follow-up has shown no evidence of disease recurrence. This report illustrates that isolated splenomegaly can be the first and only manifestation of DLBCL and emphasizes the role of splenectomy as both a diagnostic and therapeutic option in carefully selected patients.

## Introduction

Diffuse large B-cell lymphoma (DLBCL) is the predominant subtype of non-Hodgkin lymphoma (NHL), accounting for approximately 30-40% of all adult lymphomas. It is a severe cancer of mature B lymphocytes with diverse clinical presentations and has a wide range of morphological, immunophenotypic, and genetic characteristics [1,2]. DLBCL usually shows up with lymph nodes or extranodal masses that grow quickly, although primary splenic involvement is quite rare, representing less than 1% of all lymphomas and about 2% of NHLs [3,4].

Primary splenic lymphoma (PSL) is defined as a lymphoma that begins in the spleen, possibly involving the splenic hilar lymph nodes, and is diagnosed without evidence of disease in other locations at that time. Primary splenic involvement is a rare presentation of DLBCL. It is differentiated from secondary splenic involvement, which is far more prevalent and occurs in the setting of severe systemic disease [5]. Das Gupta and his team proposed diagnostic criteria stating that a lymphoma is primary to the spleen if there is no widespread nodal or extranodal illness for at least six months following splenectomy [6].

The spleen, as a lymphoid organ, is often secondarily affected in systemic lymphomas; nonetheless, primary lymphoma of the spleen poses a diagnostic challenge due to its rarity and nonspecific presentation [7]. Patients frequently exhibit constitutional symptoms such as fever, weight loss, and nocturnal hyperhidrosis, or symptoms associated with splenomegaly, including early satiety, abdominal distension, or left upper quadrant pain. In some cases, patients may not show any signs of illness, and the diagnosis of splenomegaly may be incidentally identified on imaging [8].

Radiologically, solitary splenomegaly without lymphadenopathy has a broad differential diagnosis encompassing infectious, infiltrative, and hematologic illnesses, including myelofibrosis, chronic myeloid leukemia, sarcoidosis, and storage disorders [9]. Consequently, solitary splenomegaly as a manifestation of DLBCL is both unusual and challenging to diagnose. PET-CT is crucial for distinguishing between benign and malignant causes of splenomegaly, as it detects areas of increased metabolic activity [10].

Histopathological testing subsequent to splenectomy is the definitive method for diagnosis, especially when alternative investigations, including bone marrow biopsy and peripheral smear, yield equivocal results [11]. Immunohistochemistry (IHC) further assists in the subclassification of DLBCL into germinal center B-cell (GCB) and activated B-cell (non-GCB) subtypes, each possessing unique prognostic significance and treatment responses [12].

The R-CHOP regimen (rituximab, cyclophosphamide, doxorubicin, vincristine, and prednisone) remains the major treatment for DLBCL, and it has greatly improved survival rates and the likelihood of achieving remission. The prognosis for primary splenic DLBCL, while variable, is generally favorable when the disease is localized and treated with curative intent [13,14]. This case is presented because of its rarity and diagnostic significance, underscoring the necessity of considering PSL in the differential diagnosis of isolated splenomegaly. Early detection and prompt surgical and chemotherapeutic interventions are essential for positive outcomes. This article contributes to the limited literature on primary splenic DLBCL and highlights the importance of a multidisciplinary approach for unusual hematologic presentations.

## Case presentation

**Figure 1:**
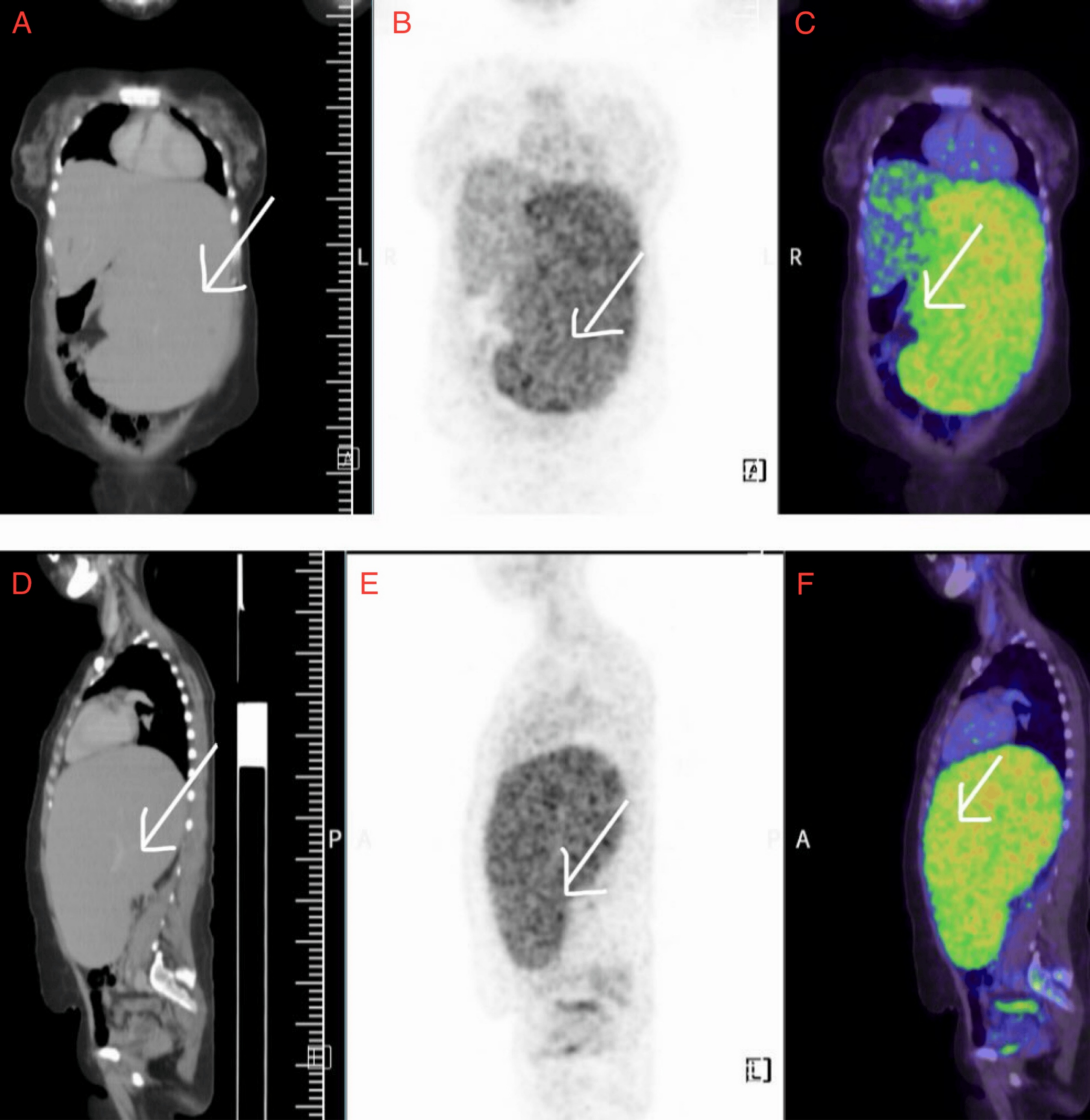
18F-FDG PET-CT revealing isolated splenomegaly without lymphadenopathy Spleen appears enlarged (splenic index-4775), crossing the midline with increased metabolic activity (SUVmax 4.12) (A) Coronal CT shows massive splenomegaly crossing midline. (B) Coronal PET demonstrates diffuse increased FDG uptake within the spleen. (C) Coronal fused PET-CT confirms metabolically active splenic enlargement (SUVmax 4.12). (D) Sagittal CT reveals marked craniocaudal splenic enlargement (splenic index ~ 4775). (E) Sagittal PET shows diffuse splenic FDG uptake. (F) Sagittal fused PET-CT confirms isolated splenic involvement with no evidence of nodal or extra-splenic disease FDG: fluorodeoxyglucose; PET: positron emission tomography; CT: computed tomography; SUVmax: highest standardized uptake value

A 42-year-old woman had increased abdominal distension and weight loss during a three-month period. She denied fever, night sweats, or anorexia, and had no prior medical conditions. On examination, she was afebrile, and her blood pressure was within normal limits. There were no signs of peripheral lymphadenopathy. The abdominal exam showed that the spleen was markedly enlarged and extended well beyond the left costal edge. The liver could not be palpated. Baseline tests, such as a complete blood count (CBC), renal and liver function tests, electrolyte testing, and viral markers, were all normal. Antinuclear antibodies (ANA) were negative. The peripheral smear showed anisopoikilocytosis, pencil-shaped cells, elliptocytes, ovalocytes, and 3% atypical cells. Abdominal ultrasound showed a very large spleen. Whole-body CT demonstrated isolated splenomegaly without lymphadenopathy. 18F-FDG PET-CT showed that the splenomegaly was metabolically active, but there was no nodal or extranodal disease, as seen in Figure 1. An initial diagnosis of hypersplenism was considered.

**Figure 2:**
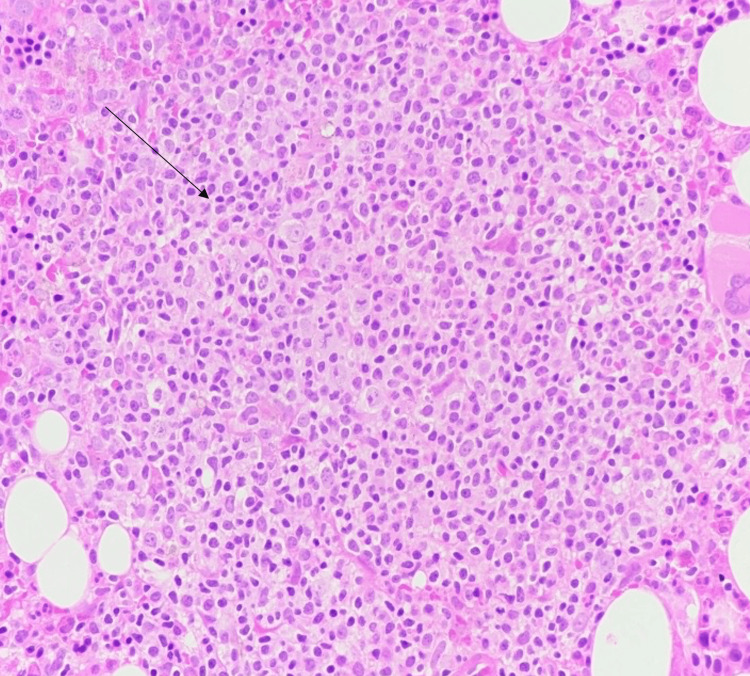
Histopathology of splenic lesion (hematoxylin & eosin stain 400x, full field view) The spleen exhibits abnormal lymphoid aggregates with a monomorphic population of lymphocytes

Bone marrow biopsy showed a myeloproliferative neoplasm consistent with pre-fibrotic stage myelofibrosis, with reactive lymphoid aggregates in the bone marrow, which were insufficient to establish a diagnosis of lymphoma. The patient then underwent splenectomy to aid in diagnosis and treatment. Histopathological examination of the spleen and hilar lymph nodes revealed diffuse large B-cell lymphoma. Microscopically, there was a monomorphic population of atypical lymphoid cells that were small to intermediate in size, as seen in Figure 2.

**Figure 3:**
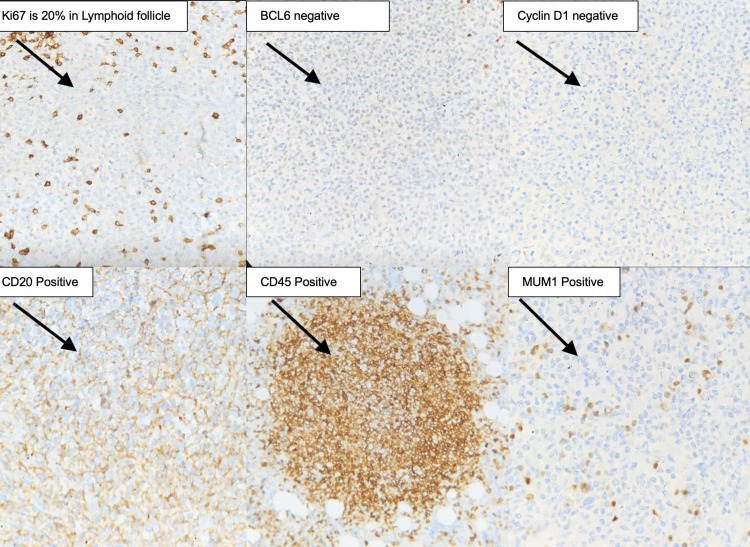
Immunohistochemical findings from the diffuse large B-cell lymphoma (all images at 400x magnification) (A) Ki67 proliferation index is approximately 20% within the neoplastic lymphoid follicles. (B) BCL6 immunostain is negative. (C) Cyclin D1 is negative. (D) CD20 is positive. (E) CD 45 Positive. (F) MUM1 is positive

Immunohistochemistry revealed diffuse large B-cell carcinoma - non-GCB subtype: tumor cells positive for CD45, CD20, BCL2, MUM1, CD3, CD5 reactive in T-lymphocytes, Tumor cells were negative for VD10, CD5, CD3, Bcl6, Cyclin D1, and CD23. CD23 was positive in preserved follicular dendritic cells; the Ki-67 index was ~60% as seen in Figure 3.

After the surgery, the patient was started on R-CHOP chemotherapy, completed three cycles, and had three additional cycles planned. She tolerated the treatment well and experienced no complications related to the regimen. A follow-up PET-CT performed two months later revealed no evidence of disease recurrence. At her most recent follow-up, the patient remained asymptomatic, with no radiological or clinical signs of recurrence.

## Discussion

Primary splenic diffuse large B-cell lymphoma (PS-DLBCL) is an uncommon subtype of NHL, constituting less than 1% of all lymphomas and approximately 1 to 2% of NHL patients. It is characterized by lymphoma confined to the spleen, with or without involvement of the splenic hilar lymph nodes, and by the absence of disease in other sites at the time of diagnosis. It can be difficult to determine whether the spleen is the true primary site in systemic lymphomas because it is more commonly involved secondarily. Our patient exhibited increased abdominal distension and weight loss over three months, without fever, night sweats, or lymphadenopathy, which is an atypical presentation for an aggressive lymphoma like DLBCL. The physical exam showed marked splenomegaly, but the blood tests were largely normal. The lack of peripheral lymphadenopathy and normal baseline investigations initially suggested hypersplenism or a myeloproliferative disease rather than malignancy. This highlights the diagnostic challenge, as PS-DLBCL frequently resembles benign or reactive splenic disorders, resulting in delayed diagnosis due to nonspecific symptoms.

Imaging investigations were crucial in guiding further evaluation. Ultrasound and CT scans showed isolated splenomegaly, while 18F-FDG PET-CT demonstrated metabolically active uptake within the spleen, strongly suggesting a malignant process. This pattern is consistent with the study by Daniel et al. (2025), which emphasized the importance of PET-CT in identifying localized splenic involvement in DLBCL and excluding systemic disease. Bone marrow examination in our patient revealed pre-fibrotic stage myelofibrosis with reactive lymphoid aggregates, which were insufficient for a definitive lymphoma diagnosis, thereby necessitating splenectomy for definitive tissue evaluation [15].

Histopathology of the splenectomy specimen confirmed DLBCL, characterized by a diffuse proliferation of atypical lymphoid cells. Immunohistochemistry showed that the tumor was positive for CD45, CD20, BCL2, and MUM1, with a Ki-67 index of approximately 60%. These findings are consistent with a non-germinal center B-cell (non-GCB) subtype. The non-GCB subtype, as noted by Daniel et al. (2025) and Wadsworth et al. (2023), is typically associated with more aggressive disease progression and a poorer prognosis than the germinal center B-cell (GCB) subtype. However, it remains highly responsive to early treatment with R-CHOP chemotherapy [15,16].

Wadsworth et al. (2023) described a case of PS-DLBCL with atypical CD30 positivity and abscess-like morphology, highlighting the histopathological heterogeneity of this entity, in contrast to the findings in our patient. Although our patient did not exhibit CD30 expression, concurrent MUM1 and BCL2 positivity supported an activated B-cell (ABC) phenotype, suggesting a more aggressive biological profile. Nevertheless, our patient responded well to combined surgical and chemotherapeutic management, reinforcing the notion that early, localized disease can overcome typically unfavorable prognostic features [16].

Splenectomy serves a dual function in PS-DLBCL, acting as both a diagnostic and therapeutic procedure. Meng et al. (2023) emphasized that splenectomy not only confirms the diagnosis but also provides symptomatic relief and cytoreduction before chemotherapy. Our patient demonstrated this dual benefit, achieving diagnostic clarity and rapid clinical improvement after splenectomy [17]. Recent studies, including Pirzada et al. (2023), have reported cases in which image-guided splenic biopsy, followed by R-CHOP without splenectomy, resulted in complete remission. This suggests a gradual shift toward less invasive diagnostic approaches. However, when imaging or bone marrow findings are inconclusive, splenectomy remains a critical option [3].

After the patient underwent splenectomy, she received six cycles of R-CHOP therapy and achieved complete metabolic remission after three cycles. This management approach aligns with current evidence. Seijari et al. (2024) reported favorable outcomes with a shortened R-CHOP regimen followed by radiation [18], whereas Pirzada et al. (2023) reported complete remission with R-CHOP monotherapy in stage I PS-DLBCL. Both studies, as well as our case, emphasize the critical role of rituximab-based chemotherapy in improving outcomes, regardless of whether splenectomy is performed [3].

The prognosis of PS-DLBCL is largely dependent on disease stage and treatment response. Localized stage I disease confined to the spleen, as observed in our patient, is associated with a favorable prognosis. Meng et al. (2023) reported that splenectomy followed by adjuvant chemotherapy results in improved long-term survival, whereas systemic disease at presentation significantly worsens prognosis. In our case, early diagnosis, localized disease, and prompt initiation of therapy facilitated the patient’s complete remission and sustained disease-free status on follow-up [17].

Isolated splenomegaly remains a diagnostic challenge. The absence of lymphadenopathy and normal laboratory results may mask underlying lymphoma. Neves et al. (2023) highlighted that the differential diagnosis of splenomegaly is broad, including hematologic, infectious, and infiltrative causes, making prompt evaluation for PS-DLBCL essential when PET-CT shows increased metabolic activity [19]. Wadsworth et al. (2023) also noted that PS-DLBCL may mimic splenic abscesses or benign cystic lesions, emphasizing the importance of histopathological confirmation [16].

Our case report also demonstrates the effectiveness of a multidisciplinary approach. Collaboration among hematology, oncology, radiology, and pathology teams enabled accurate diagnosis and improved treatment outcomes. Meng et al. (2023) and Seijari et al. (2024) both highlighted the importance of coordinated care in rare lymphoid malignancies, where standard treatment protocols are limited. In summary, our case of PS-DLBCL presenting as isolated splenomegaly supports findings from recent studies. Although PS-DLBCL is rare and often challenging to diagnose, it can achieve complete remission if identified and treated promptly with splenectomy and R-CHOP chemotherapy. This case contributes to evidence that primary splenic DLBCL has a favorable prognosis when diagnosed early and managed by a specialized multidisciplinary team, despite its biologically aggressive nature.

## Conclusions

PS-DLBCL is an extremely rare subtype of NHL that may present solely as isolated splenomegaly, often mimicking benign or reactive conditions. This report underscores the importance of maintaining diagnostic vigilance when evaluating unexplained splenomegaly. Splenectomy provides both diagnostic and therapeutic benefits, supplying tissue for histopathological confirmation while relieving hypersplenism. The subsequent treatment with R-CHOP chemotherapy led to complete remission, illustrating the effectiveness of combined surgical and chemotherapeutic management. Early detection, timely intervention, and a multidisciplinary approach are critical for improving prognosis. Reporting such rare cases enhances clinical understanding and helps refine diagnostic strategies for atypical lymphoid malignancies.
